# Watching Nature Videos Promotes Physiological Restoration: Evidence From the Modulation of Alpha Waves in Electroencephalography

**DOI:** 10.3389/fpsyg.2022.871143

**Published:** 2022-06-07

**Authors:** Simone Grassini, Giulia Virginia Segurini, Mika Koivisto

**Affiliations:** ^1^Department of Social Studies, University of Stavanger, Stavanger, Norway; ^2^Department of Psychology, Norwegian University of Science and Technology, Trondheim, Norway; ^3^Department of Psychology, University of Turku, Turku, Finland

**Keywords:** EEG, EDA, nature, restoration, cognition

## Abstract

Various lines of evidence have shown that nature exposure is beneficial for humans. Despite several empirical findings pointing out to cognitive and emotional positive effects, most of the evidence of these effects are correlational, and it has been challenging to identify a cause-effect relationship between nature exposure and cognitive and emotional benefits. Only few of the published studies use psychophysiological methods to assess the biological correlates of these positive effects. Establishing a connection between human physiology and contact with natural settings is important for identifying cause-effect relationships between exposure to natural environments and the positive effects commonly reported in connection to nature exposure. In the present study, we recorded physiological indexes of brain activity (electroencephalography) and sympathetic nervous system (electrodermal activity), while the participants were presented with a series of videos displaying natural, urban, or neutral (non-environmental, computerized) scenes. Participants rated the scenes for their perceived relaxing value, and after each experimental condition, they performed a cognitive task (digit span backward). Participants rated natural videos as the most relaxing. Spectral analyses of EEG showed that natural scenes promoted alpha waves, especially over the central brain. The results suggest that experiencing natural environments virtually produces measurable and reliable brain activity markers which are known to be related to restorative processes.

## Background

The literature on environmental psychology has defined a restorative environment as one that can help to restore drained emotional and/or cognitive resources. Outdoor environments that feature natural elements have been shown to effectively promote restorative effects (Hettinger, 2012). Human exposure to natural settings has been shown to be associated to psychological and psychophysiological positive effects, such as stress reduction, improvement of moods and mental health (as, e.g., reducing symptoms of anxiety and depression – see Grassini, 2022 for a review), and cognitive recovery from task overload (Ulrich, 1979; Kaplan and Kaplan, 1989; Berman et al., 2008; Hartig et al., 2014).

A long line of empirical evidence has shown that natural settings can promote stress reduction and ease physiological arousal (Laumann et al., 2003; Stigsdotter and Grahn, 2004; Tyrväinen et al., 2014). Placing natural elements in urban areas has been shown to improve mental health (Tzoulas et al., 2007), and natural elements indoor has been shown to promote psychological well-being (Raanaas et al., 2016). In addition, viewing nature from a window helps faster recovery from physical exercise (Engell et al., 2020). The positive effects of nature have been extensively studied in the context of work well-being, and it has been shown that interactions with natural settings may be beneficial to reduce work-related stress (Korpela and Kinnunen, 2010; Korpela et al., 2015).

Current scientific literature has extensively investigated the ability of natural settings to restore attentional and cognitive abilities. Current theories of the cognitive effects of exposure to natural stimuli (see Kaplan and Kaplan, 1989; Kaplan, 1995) propose that cognitive abilities are restored by natural settings, and therefore predict that after the exposure to a natural environment (compared, e.g., to an urban one), people would perform better in cognitive and memory tasks. Several lines of evidence have indicated that natural settings have the capacity to restore human attentional and cognitive abilities (for reviews, Ohly et al., 2016; Stevenson et al., 2018). Among cognitive skills, being exposed to natural elements has been shown to increase performance in working memory tasks (Bratman et al., 2015), improve performance in sustained attention exercises (Berto, 2005), increase cognitive control, and concentration (Hartig et al., 2003; Berman et al., 2008, 2012).

The mechanisms underpinning the restorative effect of nature for humans have been extensively studied (Berto, 2005; Haga et al., 2016; Joye et al., 2016). A widely reported theoretical point of view is that humans have an innate tendency to experience positively the contact with natural environments. In this context, the Biophilia hypothesis is often cited (Wilson, 1984). According to this hypothesis, humans are biologically prepared to positively respond to the environments with positive attributes for biological survival (see also Joye and Van den Berg, 2011). Some studies have found that some specific features of natural stimuli are related to restorative effect. Images of nature were shown to be rated as more restorative than images of other types of environments (Berto, 2005). Factors other than physical stimulus features have been also studied regarding their restorative effects (Haga et al., 2016; Egner et al., 2020). A number of different types of auditory and visual stimuli have been found to be perceived as restorative. Natural sounds have been shown to help restore mental fatigue (Ratcliffe et al., 2013; Benfield et al., 2014; Emfield and Neider, 2014; Jahncke et al., 2015).

The empirical findings of the benefits associated to the exposure to natural environments are often theoretically explained with two prominent theories (Hartig et al., 1996). The first theory, the Stress Reduction Theory (SRT; Ulrich, 1983; Ulrich et al., 1991) put emphasis on psycho-physiological stress, while the other one, the Attention Restoration Theory (ART; Kaplan and Kaplan, 1989; Kaplan, 1995), focuses on the cognitive recovery from directed attention fatigue. The SRT (Ulrich, 1983; Ulrich et al., 1991) underlines the physiological effect of the exposure to natural settings. The theory suggests that qualitative features commonly found in natural settings may directly support psychophysiological stress recovery, and this may occur because nature elicits positive effects. The SRT finds its background on the theories of human evolution: humans may be biologically prepared to optimally respond to environmental features that imply possibilities for biological survival, and therefore there may be an evolutionary basis for aesthetic and restorative responses to some types of natural scenes. Such theoretical approach is connected to the Biophilia hypothesis (Wilson, 1984). The ART (Kaplan and Kaplan, 1989; Kaplan, 1995) put its emphasis on the ability of natural environments to promote cognitive restoration and on the positive effect of natural settings on human attentional fatigue. Researchers have proposed that these two theories may not be mutually exclusive and that they complement each other (Hartig et al., 2003), as several studies have found attentional fatigue and stress to be linked (Cohen and Spacapan, 1978; Ulrich et al., 1991; Kaplan, 1995; Lepore and Evans, 1996). However, it has also been argued (see, e.g., Egner et al., 2020) that the attention restoration produced by nature may be a secondary effect, primarily dependent on the relaxation.

A considerable number of studies have examined human health and cognitive functions associated with viewing images, videos, and other simulations of nature (Ohly et al., 2016; Stevenson et al., 2018), including in recent years, highly immersive virtual reality (Browning et al., 2020; Yeo et al., 2020). However, only a handful of studies have attempted to understand the brain dynamics in response to natural or non-natural settings. Kim et al. (2010) employed functional magnetic resonance imaging (fMRI) to examine the brain areas activated in response to natural or urban images. Their results revealed that perception of urban images was associated with an enhanced activity in the amygdala, an area known to be associated with impulsivity (Gopal et al., 2013; Kerr et al., 2015), anxiety (Hahn et al., 2011; Kim et al., 2011), and stress (Veer et al., 2011; Sripada et al., 2012). The perception of natural scenes was found to be associated with an increased activity in the anterior cingulate and insula areas (Kim et al., 2010). These brain areas were found by previous studies to be related to positive social behavior (e.g., empathy and altruism, see Fan et al., 2011; Lamm et al., 2011). Neurophysiological experiments have shown that exposure to natural environments stimulate brain activity related to tranquility (Hunter et al., 2010).

Several studies have used electroencephalography (EEG) to examine brain functional dynamics related to the experience of natural settings in different frequency bands. The frequency bands are delta (0.5–4 Hz), theta (4–8 Hz), alpha (8–13 Hz), beta (13–30 Hz), and gamma (>30 Hz) bands. The alpha activity has received most attention because it is related to relaxed state of mind. The alpha waves are understood to be produced from postsynaptic potentials in a neural network involving the dorsal anterior cingulate cortex, the anterior insula, the anterior prefrontal cortex, and the thalamus that is associated with alertness and attention (Sadaghiani et al., 2010). It has been suggested that such brain network is responsible for internalized alertness (Coste and Kleinschmidt, 2016) and is associated with variations in involuntary attention (Dosenbach et al., 2008). It has been proposed that alpha waves recorded using EEG may be indexes an inhibitory mechanism generated by a thalamic-cortical loop (Foxe and Snyder, 2011). Several previous studies have found alpha waves to be modulated while experiencing natural environments. An increase in alpha power in the EEG (8–13 Hz) has been found to be a marker of the experience of natural vs. urban photographs (Ulrich, 1981; Chang et al., 2008; Grassini et al., 2019). It has also been suggested that the experience of natural environments is able to modulate brain activity associated with cognitive-inhibitory mechanisms, and that this effect is reflected by an increase of alpha brain activity markedly over the brain’s central areas (Ulrich, 1981; Grassini et al., 2019). A recent review (Norwood et al., 2019) reported several studies that connected higher alpha over frontal lobes to the experience of natural environments or other environmental settings perceived as positive (Chang and Chen, 2005; Choi et al., 2015; Chiang et al., 2017; Bailey et al., 2018).

The physiological basis of the beta rhythms has been long being disputed. It has been proposed that beta waves are generated in basal ganglia and thalamic structures (Bevan et al., 2002; Leventhal et al., 2012). Later studies have shown that beta waves may emerge within internal dynamics of the neocortex that are dependent on extrinsic synaptic drive originating in other structures as basal ganglia and thalamus (Sherman et al., 2016). Beta oscillations have been shown to be associated to the activity of the motor cortex, to sensory perception, and to selective and spatial attention (Neuper and Pfurtscheller, 2001; Siegel et al., 2008; Jones et al., 2010; Miller et al., 2012). Grassini et al. (2019) found that while viewing nature images increased alpha activity, the beta activity was at the same time decreased, suggesting that less attentional resources were used during viewing nature images, compared to urban ones.

Whereas delta and gamma bands have not received practically any attention in studies on the psychological effects of nature, there is some evidence that theta band activity may be modulated during viewing natural stimuli. Theta activity has been associated with cognitive control and error processing, and an origin of increase theta power has been found in the anterior cingulate cortex (Cavanagh et al., 2010, 2012; Cavanagh and Shackman, 2015). Sahni and Kumar (2020) reported that viewing nature videos increased both theta and alpha power, suggesting that natural stimuli provoke a relaxed but alert state of mind.

Other studies have employed EEG as method to estimate the positive emotions related to the experience of natural settings (Roe et al., 2013; Aspinall et al., 2015), and their findings confirmed that natural environments promote more positive emotions and higher level of relaxation. However, these latter studies did not directly analyze the EEG data but relied on the proprietary algorithms of the EEG manufacturer for data analysis, and therefore is not possible to understand whether alpha waves were modulated by their experimental conditions.

Non-brain physiological indexes such as heart rate (HR; see Annerstedt et al., 2013; Yu et al., 2018), heart-rate variability (HRV; see Park et al., 2007, 2010; Gladwell et al., 2016), and electrodermal activity (EDA, sometimes referred in the literature as skin conductance or galvanic skin responses, Alvarsson et al., 2010; Hedblom et al., 2019; Browning et al., 2020) have been also used to understand the human physiological response to the exposure to natural settings.

Heart rate variability (HRV) is defined as the variation in the intervals between heartbeats. Such physiological index is often used as an indicator of the activity of the autonomic nervous system (ANS). Higher HRV has been linked with an increased adaptability of the ANS and has been proposed to be associated with positive health factors (Brown et al., 2013). Several studies have showed that HRV is related to stress and that different components of HRV can be linked to stressors (for a review see Kim et al., 2018). Previous studies have shown that exposure to natural environments increases HRV (Park et al., 2007, 2010; Gladwell et al., 2016), suggesting that nature exposure is accompanied with reduced stress and enhanced relaxation.

Electrodermal activity is controlled by the sympathetic nervous system, and it is modulated by the activity of the skin sweat glands. EDA is often used to measure psychological or physiological arousal (Critchley, 2002). Previous studies have found that EDA is reduced when people are exposed to natural settings (Alvarsson et al., 2010; Hedblom et al., 2019; Elsadek et al., 2020). However, the inverse effect has been also found (Browning et al., 2020), suggesting that other variables or experimental factors may be contribute to the modulation of EDA in relation to the exposure to natural settings.

A small number of studies have considered the use of more than one physiological measure to cross-validate their results across different methods. The recently published study of Elsadek et al. (2020) analyzed physiological activity using EDA and EEG on people looking outside a window facing urban or green spaces. It found a higher level of alpha waves over the frontal and occipital brain areas, and a decrease in skin conductance level. However, this study only offered a limited spatial resolution on the brain data, due to the low-density EEG equipment using only four electrodes (two frontal and two occipital). This choice is probably attributable to the electrode positions provided by the Emotiv EPOC equipment that was used in the study, but it comes at odd with the reported localization of alpha increase during nature exposure, which was revealed to be prevalently over the central brain areas in the studies of Ulrich (1981) and Grassini et al. (2019).

One limitation that is shared by many of these studies is the lack of a control or neutral condition when comparing the influence of natural and urban environments. The studies that focus on comparing urban and natural environment (see, e.g., Ulrich, 1981; Aspinall et al., 2015; Grassini et al., 2019; Elsadek et al., 2020) generally fail to answer the question whether the natural or the urban stimuli are responsible for the reduction/or increase of physiological arousal (e.g., indexed with EDA) and modulation of cognitive mechanisms (e.g., indexed using EEG).

The present study aimed to use both brain (EEG) and non-brain (EDA) indexes (together with subjective evaluations) to understand physiological dynamics of the interaction with natural settings. This was done in a laboratory setting, where videos of nature (without audio) were showed to the participants while biological signals were recorded. Furthermore, we attempted to understand whether urban environments or natural environments were responsible for an eventual modulation of physiological brain and non-brain activity by adding control condition to the experiment. Our hypotheses were that the vision of nature video would modulate brain early alpha waves recorded over the central brain areas as shown in previous lab-controlled studies employing static images (Ulrich, 1981; Grassini et al., 2019). We did not have a hypothesis about whether nature or urban environment would be responsible of such effect when compared to a neutral stimulation. We expected EDA to be reduced when watching nature videos, but we had no preliminary hypothesis about whether the effect would be more pronounced versus neutral or urban videos.

## Materials and Methods

### Participants

Twenty-four neurologically healthy students with normal or corrected-to-normal vision participated in the study (mean age = 24.8, SD = 5.1, range = 19–36 years, ten men). They were selected from the pool of participants from the introductory psychology courses in University of Turku. The students received course credits as reward for their participation. The experiment was conducted in accordance with the Declaration of Helsinki and with the understanding and written consent of each participant and was approved by the Ethics Committee for Human Sciences at the University of Turku. Before the experimental session, the participants were asked to read an information sheet on the experiment, to read and sign the informed consent, and to self-assess their handedness. Only students reporting to be right-handed were tested. The sample size was determined based on the observed effect and n from previously published EEG studies on brain activity in response to environmental exposure (Grassini et al., 2019). The number of participants recruited for our study is also in line with recent investigations on the relationship between participants number and EEG data reliability for relatively long tasks during EEG recording (Vozzi et al., 2021). Data from two participants were excluded from the analysis of cognitive task (digit-span backward scores), and two were excluded from the analysis of the EDA data due to technical issues in data collection.

### Stimuli and Procedure

**FIGURE 1:**
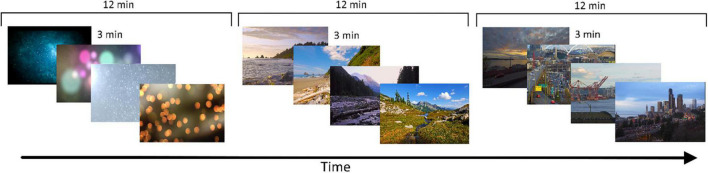
Example of the experimental procedure. In this example, four videos of the neutral condition are followed by four videos of the nature conditions, and four videos of the urban condition concluded the experimental session. Each condition was composed of four 3-min videos.

The stimuli consisted of videos depicting natural, urban, or neutral settings (referred hereinafter as the three experimental conditions). A total of nine videos per condition were selected by the study authors. Every participant was exposed to four consecutive videos of the same environment type (natural, urban, or neutral) before passing to four consecutive videos of another environment, and four consecutive videos of the last environment. The four videos for each environment type were selected randomly among the nine available videos. The order of the experimental conditions (i.e., environment type) was randomized across participants. Every video lasted for 3 min, for a total of 12 min of exposure per each experimental condition. Natural settings videos depicted a variety of natural environments, among those, mountain lakes, beaches, forest, and fields. Urban environment videos showed city-like landscapes, and depicted for example traffic, buildings, and other types of common environments displaying urban or industrial buildings. Urban videos contained eventually some natural elements (e.g., threes on the edges of a street, or a city seaside view). Natural videos were selected appositely to avoid any type of non-natural element. The videos were selected from material retrieved from the internet. The authors SG and GS selected independently a list of suitable videos, and together reviewed and chose those that were judged as the most appropriate for the present study. Criteria that were used to selecting the videos were: high enough quality of details, absence of numbers/words in the environment, absence of close-ups of people/faces. Furthermore, markedly unpleasant urban environments were avoided, trying not to suggest a strong judgment bias to the participants. For the neutral setting condition, lines depicting slowly changing colorful shapes or patch of colors were selected, alike those often used in computer screensavers. All videos were displayed with a resolution of 1920 × 1080 pixels, and a frame rate of 24 frames/second, and were viewed at around 2 m of distance on a 17-inch LCD screen. After each video, the word “relaxing” was presented on screen and the participants rated how relaxing (from 1 to 9; 1 = not at all, 9 = very much) they found the video. The “relax” score for each condition was then averaged across videos of the same type for each participant. The experiment was run using E-prime 2. Figure 1 shows an exemplification of the stimuli used in the experiment and the experimental procedure.

After each condition (i.e., block of four-videos), the participants were asked to perform a digitalized visual version of the Digit Span Backward Task (DSB). After the participants had been instructed to the task, they were asked to perform the DSB before the beginning of the experiment. This was done to allow the participants to familiarize with the task. In the DSB, a series of digits were displayed on the screen, following one another. The digit displayed were randomly chosen from 1 to 9. Participants were instructed to recall the digits backward (e.g., if “3, 4, 5” were presented, the subject had to press the keys “5, 4, 3” using the keyboard). In the case the task was successful for two times in a row, participants were then asked to perform the task again with a longer list of digits. The task started with only 2 digits, and the participants could go to indefinitely long list of digits according with their performance. The digit list was made longer until the point when participant failed to inversely recall the list for two succeeding trials. The score for each participant was calculated on the length of the longest list the participant successfully performed. The DSB task aimed to detect cognitive restorative effect from the exposure to the videos (working memory). Several studies have implemented this type of cognitive task to assess the restorative effect arising from the exposure to natural settings (Ohly et al., 2016 for a review). The subjects were presented with a computerized, visual version of the DSB [similarly to the one used in Grassini et al. (2019)], and participants were instructed to perform the task using a keyboard. However, it is worth nothing that the commonly used DSB task generally uses vocal stimuli and verbal response recall. Previous studies have also used a computerized version of the DSB to successfully assess working memory (AuBuchon et al., 2015).

### Electroencephalography

Electroencephalographic brain activity was assessed at 64 sensors placed according to the international 10–10 electrode system. The electrodes were placed on the head using a sintered Ag/AgCl cap featuring active electrodes (Easycap GmbH, Herrsching, Germany). NeurOne 1.3.1.26 software and Tesla #MRI 2013011 and #MRI 2013012 amplifiers (Bittium Corporation, Oulu, Finland) were used for data collection. Signal was referenced online to Cz, and the ground electrode was placed on position AFz. The recording sampling rate was set to 500 Hz.

Electroencephalography data was processed offline using MATLAB (v. R2019a; The MathWorks, Inc., Natick, MA, United States) and with the EEGLAB toolbox version v2021.0 (Delorme and Makeig, 2004). Intervals between the videos (where the participants were asked to answer a question on their subjective evaluation of the latest video) were removed from the recorded signal. Data was high-pass filtered at 1 Hz with a Hamming windowed-sinc FIR filter (“pop_eegfiltnew”). Line noise was removed using the cleanLineNoise function included in the PREP pipeline toolbox v. 0.55.4 (Bigdely-Shamlo et al., 2015). Artifactual data in the continuous EEG recording and bad electrodes were then automatically attenuated or removed using the Artifact Subspace Reconstruction method implemented in the Clean Rawdata plug-in (“clean_rawdata,” Kothe, 2013; Piazza et al., 2016). Removed electrodes were interpolated using spherical interpolation, and data was then re-referenced to average. EEG data was submitted to extended Infomax independent component analysis (ICA, “pop_runica.m”) to detect and afterward remove non-neural activity (eye and muscle movements). An automatic component classifier method (Multiple Artifact Rejection Algorithm: MARA; Winkler et al., 2014) was used to automatically identify and eliminate artifactual independent components.

The EEG data pre-processing pipeline was created adapting the suggestions reported in Miyakoshi (2021). Power Spectra Density (PSD) was computed using the EEGLAB spectopo function (pwelch, see Welch, 1967), and the data was analyzed over segments of 2 s, with a 50% overlap between segments (hamming window, zero-padding to 1024 data-point epochs length). Average values of PSDs for each of the experimental conditions, for all the EEG electrodes, and for all the frequencies from 2 to 30 Hz were obtained, to be able to have an overview of the activity for delta, theta, alpha, and beta waves.

### Electrodermal Activity

Electrodermal activity was measured using an electrode couples connected (long finger and index finger on the palmar side) to a wireless handcuff placed on the left, non-dominant hand. The recording baseline was individually adjusted using the Biopac default function for each participant prior to the experiment. The equipment used was the Biopac MP150 recording system (Biopac Systems, Inc., Goleta, CA, United States) and the amplifier BN-PPGED-R with 16-bit resolution and a sampling rate of 2 kHz. Participants were asked to avoid movements and to keep the left hand as still and relaxed as possible. The EDA data was operationalized computing two different variables exploring different aspects of the activity of sympathetic nervous system, the skin conductance level (SCL), and the number of skin conductance responses (SCRs). The SCL shows tonic and long-lasting activity of the sympathetic nervous system, while SCRs indicate phasic activity reflecting the number of stimulated nerve impulses regulating the secretion of sweat (Benedek and Kaernbach, 2010). SCRs were analyzed using the MATLAB Ledalab toolbox (Ledalab 3.4.9; Benedek and Kaernbach, 2010). Signal was first down-sampled (factor mean function) to 10 Hz, and then analyzed using continuous decomposition analyses (extraction of continuous phasic/tonic activity). The number of SCRs (during each of the videos) were then computed (amplitude threshold for inclusion of phasic responses = 0.01 μS), and absolute values were then exported for each participant. Average tonic SCL amplitude for each of the experimental conditions was also exported (μS). Results for videos of the same category (natural, urban, neutral/control), where averaged together, to obtain a unique index per each condition and for each participant.

### Statistical Analyses

Electroencephalography results were analyzed using the data analysis package integrated in the EEGLAB study function. The difference between the three experimental conditions were first analyzed for the early alpha waves (8–11 Hz), as brain waves at these frequencies were found to be the most affected by the presentation of natural vs. non-natural sceneries in static photos (Grassini et al., 2019). The differences in the low alpha waves (PSD) were analyzed for all the electrodes. In computing statistic for the scalp topography, the presented exact *p*-values were obtained with parametric ANOVAs, while multiple comparisons were corrected with spatial clustering, using the traditional threshold of significance level of *p* < 0.05 (method: triangulation, cluster statistic: maxsum) as provided in the Matlab toolbox Fieldtrip (Fieldtrip Lite v20210330). Repeated-measures ANOVA was computed using experimental conditions (3) as factor, and *post hoc t*-tests were computed to study differences of activity in low alpha between the experimental conditions.

To control for the specificity of the effect for early-alpha waves (as reported in previous studies, see, e.g., Grassini et al., 2019), a further analysis in the wave-spectral domain was performed for two cluster of electrodes (central and occipital) that were reported to show prominent effects in processing. Therefore, PSD values for the electrodes placed over the center-frontal (electrodes FCz, FC1, and FC2 – central cluster) and center-occipital (electrodes Oz, O1, and O2 – occipital cluster) were averaged for each participant separately for the three experimental conditions, and the values compared over the spectral domain using an ANOVA and *post hoc t*-tests.

IBM SPSS v. 26 was used for the rest of the statistical analyses. Data from the subjective evaluations of the videos were analyzed using a repeated-measures ANOVA comparing the mean relaxation ratings between the three different experimental conditions, the statistically significant effects followed by pair-wise comparisons with two-tailed *t*-tests. Analyses of EDA and DSB were computed in the same way. For these analyses, when the data sphericity assumption was violated, Greenhouse–Geisser corrected *p*-values were reported.

## Results

### Subjective Ratings

**FIGURE 2:**
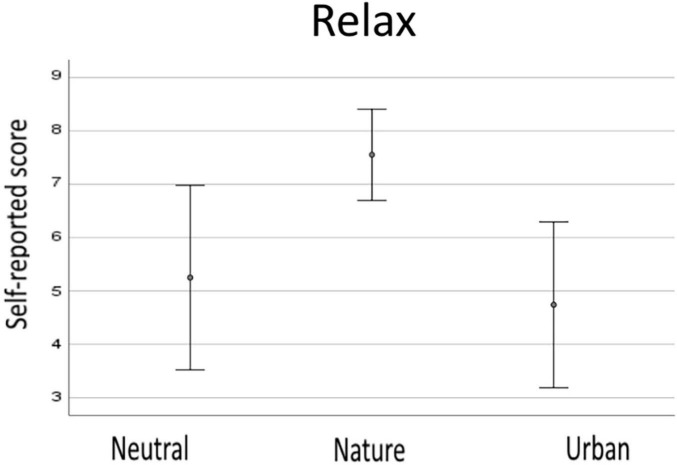
Descriptive data from subjective responses for neutral, nature, and urban experimental condition. Error bars show standard deviations (SD).

The relaxation ratings given after each of the four videos/condition were averaged to obtain a single score for each of the three experimental conditions. The scores ranged from a minimum of 1 to a maximum of 9. Figure 2 shows the descriptive results for behavioral data. The one-way ANOVA showed that the perception of relaxation differed between the conditions, *F*(2,46) = 43.05, *p* < 0.001, e2 = 0.652. *Post hoc t*-tests revealed that natural environment videos were rated as the most relaxing and differed statistically both from the neutral (*p* < 0.001) and urban (*p* < 0.001) ones. Ratings did not differ between neutral and urban environments (*p* = 0.184).

### Digit Span Backward Task

**FIGURE 3:**
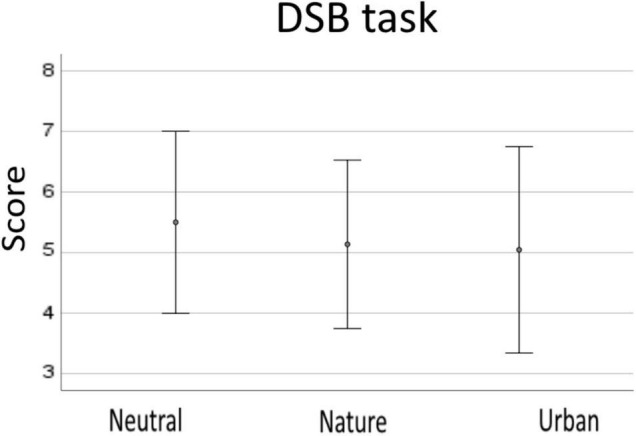
Descriptive data from the DSB task for neutral, nature, and urban experimental condition. Error bars show standard deviations (SD).

ANOVA analysis showed that there were not statistically significant differences between the DSB task scores between the experimental conditions (*p* = 0.189). Descriptive data are reported in Figure 3.

### Electroencephalography

**FIGURE 4:**
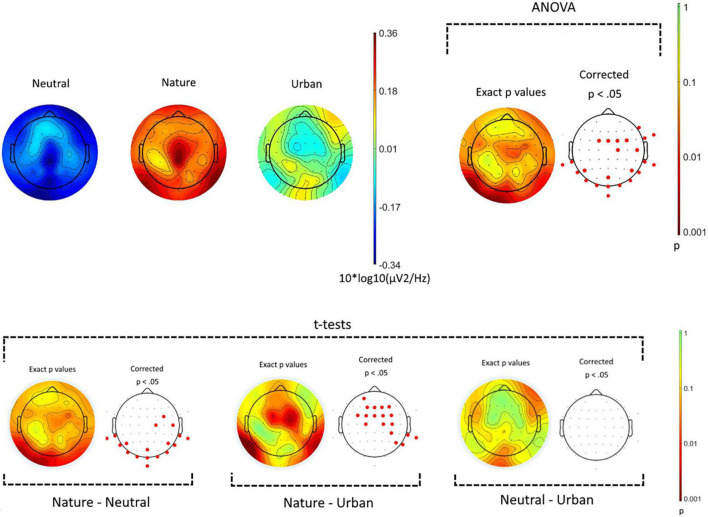
Spatial distribution of power spectral density for early-alpha frequency band (8–11 Hz). The top panel shows the power spectral density for the three experimental conditions (neutral, nature, and urban videos). On the top-right side, the results from the ANOVA showing in red the electrodes where *ps* < 0.05. The bottom panel show the results of the *post hoc t*-tests.

Oscillatory activity during the display of the videos (3) were compared for every recorded electrode (64), and for the early-alpha frequency band (from 8 to 11 Hz). Figure 4 (top panel) shows raw values and the results from the one-way ANOVA comparing the three experimental conditions. Visual inspection of the raw scores revealed that in low alpha waves frequencies, brain activity was generally higher across all the scalp during the videos displaying natural environments, compared with other conditions, while the neutral environment videos promoted the lowest level of early-alpha across all the scalp areas. Statistical analyses showed that the three experimental conditions differed, in the low alpha spectrum, over occipital-temporal and central sensors. *Post hoc t*-tests (Figure 4 bottom panel) showed that videos of natural environments enhanced the production of early-alpha waves vs. neutral videos (over occipital-temporal areas), and vs. urban videos (over central-frontal areas). No statistically significant differences were revealed for any electrodes between neutral and urban conditions.

**FIGURE 5:**
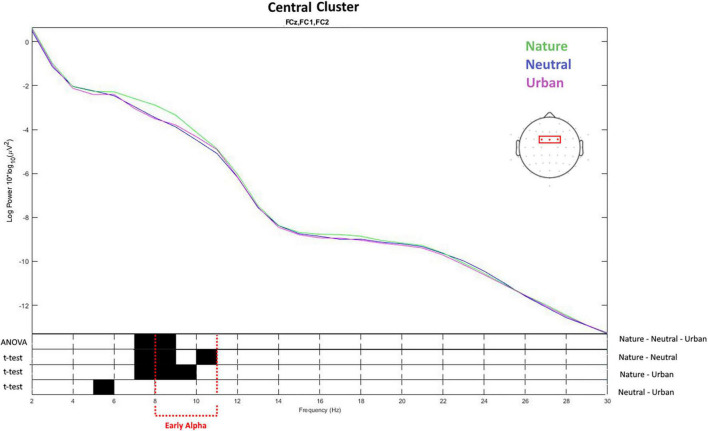
The top of the figure presents the values of spectral power obtained averaging the values for the electrodes placed over the central brain area. The frequency (*X*-axis) and spectral power (*Y*-axis) for the three experimental conditions (neutral, nature, and urban videos). The bottom of the figure shows the results of ANOVA and *t*-tests on the differences between the conditions on the spectral power for each frequency from 2 to 30 Hz. The black color shows the frequency values in which the spectral power values were statistically different between the conditions.

**FIGURE 6:**
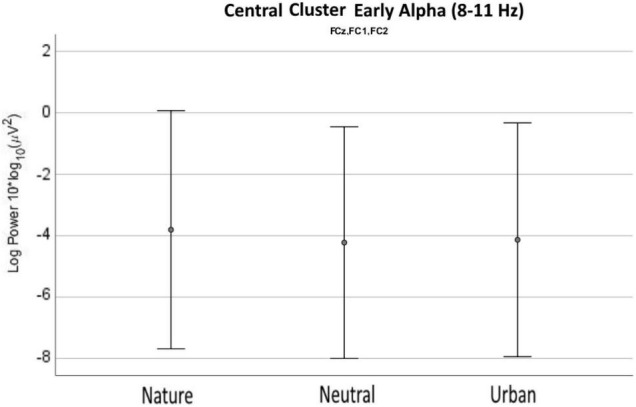
Early alpha waves power density values as recorded (averaged) over the central cluster for neutral, nature, and urban experimental conditions. Error bars show standard deviations (SD).

Based on the differences revealed by the ANOVA in the previous analyses, two electrode cluster were individuated (central and occipital) and the signal was analyzed in the spectral domain from 2 to 30 Hz. This analysis aimed to study whether the effects of viewing nature videos on spectral power was restricted specifically to the lower alpha band or whether they could be observed also in other frequencies. For the central cluster (Figure 5), the ANOVA showed that the three experimental conditions differed in early alpha (8–9 Hz) as well as in late theta (7–8 Hz) activity. Nature videos increased waves both in the early alpha and late theta, as compared with neutral and urban videos. Urban and neutral conditions differed only in slow theta frequencies between 5 and 6 Hz. Average values and distribution are shown in Figure 6.

**FIGURE 7:**
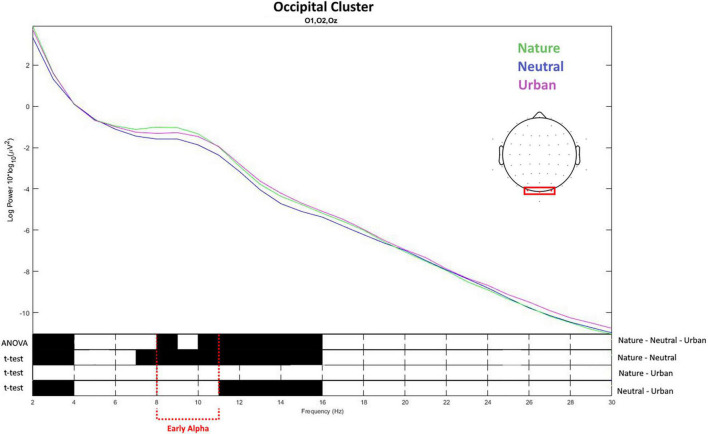
The top of the figure presents the values of spectral power obtained averaging the values for the electrodes placed over the occipital brain area. The frequency (*X*-axis) and spectral power (*Y*-axis) for the three experimental conditions (neutral, nature, and urban videos). The bottom of the figure shows the results of ANOVA and *t*-tests for the difference between the conditions on the spectral power for each frequency from 2 to 30 Hz. Black color shows the frequency values in which the spectral power values were statistically different between the conditions.

**FIGURE 8:**
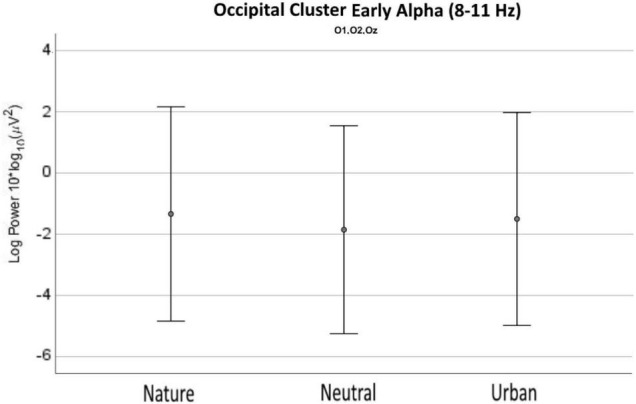
Early alpha waves power density values as recorded (averaged) over the occipital cluster for neutral, nature, and urban experimental conditions. Error bars show standard deviations (SD).

For the occipital cluster (Figure 7), ANOVA showed that the three experimental conditions differed in early alpha (from 8 up to 11 Hz) as well as in early delta (2–4 Hz), and late alpha (11–13 Hz) up to early beta waves (13–15 Hz). Nature videos promoted higher alpha and early theta waves vs. the neutral videos in early delta (2–4 Hz), late theta (7–8), early and late alpha (8–13 Hz), as well as in early beta (13–15 Hz). No differences were found between nature and urban conditions. Urban videos showed to promote early-delta (2–4 Hz), late-alpha (11–13 Hz), and early-beta waves (13–15 Hz) compared to neutral ones. Average values and distribution are shown in Figure 8.

### Electrodermal Activity

**FIGURE 9:**
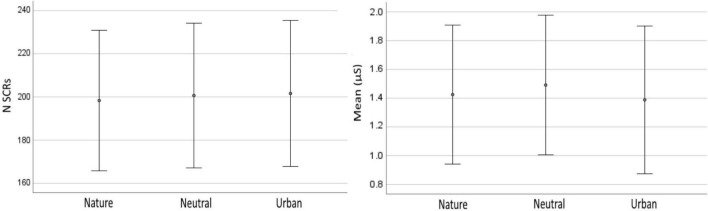
Electrodermal activity in neutral, nature, and urban experimental conditions. Error bars show standard deviations (SD).

The number of SCRs recorded for each of the experimental conditions were averaged and a singular numerical score was obtained for each condition. EDA – SCR scores were of 198.33 (SD = 32.57) for nature videos, 200.65 (SD = 33.53) nSCR for neutral videos, and 201.61 (SD = 33.86) for urban videos. One-way ANOVA showed that phasic activity SCRs did not differ between the three types of videos, *p* = 0.068 (Figure 6, left chart). Data for the tonic EDA (SCL) was averaged and analyzed in the same way as that for SCRs. EDA – SCL scores were of 1.43 (SD = 0.48) μS for nature videos, 1.49 (SD = 0.49) μS for neutral videos, and 1.39 (SD = −51) for urban videos. One-way ANOVA detected a statistically significant difference between the experimental conditions, *F*(2,42) = 3.45, *p* = 0.041. *Post hoc t*-tests, revealed that the lowest tonic activity was recorded in response to urban videos, and it statistically differed from the tonic activity recorded during the vision of the neutral videos (*p* = 0.025) but not from tonic activity related to the vision of nature videos (*p* = 0.256). Nature videos produced the second lowest level of EDA-SCL, but it did not differ from the one associated with neutral videos (*p* = 0.146). Data averages and distribution is shown in Figure 9.

## Discussion

Even though it is widely acknowledged in the scientific literature that natural environments provide restoration and promote positive feelings (Kaplan and Kaplan, 1989; Hartig et al., 2014), it is still unknown how this exactly happens, or which biological processes are mediating the restorative effects.

The recently published studies of Grassini et al. (2019) and Elsadek et al. (2020) showed that exposure to natural settings, either real or in digitalized form, modulates brain activity and non-brain physiological responses, in line with results from older studies (Ulrich, 1981). Importantly, the reported EEG data pointed out that exposure to natural setting may be associated to an increase in brain alpha waves (especially early-alphas), that was hypothetically attributed to a down-modulation of attentional and cognitive processes (Grassini et al., 2019). Other studies have found that alpha-waves are associated to the experience of environments with positive values (Chang and Chen, 2005; Choi et al., 2015; Chiang et al., 2017; Bailey et al., 2018).

The present study aimed to confirm previous findings using more immersive scenes compared to some of the previous studies (Ulrich, 1981; Chang et al., 2008; Grassini et al., 2019). Furthermore, the present study aimed to compare EEG and EDA activity in response to nature environment not only to urban environments (as commonly done in this field of research), but also to a control condition. In this way we attempted to understand if the effect of modulation of physiological arousal (measured using EDA) and EEG early alpha waves were modulated for the natural or for the urban environment compared to the control condition.

In the present experiment we recorded EEG and electrodermal activity while the participants viewed passively a series of scenes from natural, urban, or “control” environments. The control environment was obtained using computerized non-realistic scenes. After viewing each video, the participants were asked to self-report how relaxing they found the video. In line with several previous findings, participants rated as more relaxing the presentation of natural environments compared to those of urban environments. Ratings of perceived relaxation did not differ between neutral and urban environments video.

Our results are in line with our prediction concerning the modulation of early-alpha waves during the experience of videos of natural environments. The distribution of the early-alpha waves modulation showed to be mainly over central brain areas, in line with the findings of Ulrich (1981) and Grassini et al. (2019). The analyzed central early-alpha activity in response to the control condition videos did not differ from the one in urban videos, arguing for the early-alpha wave effect to be driven by the nature condition and not by the urban one. This finding may have important theoretical and practical implication: the scientific literature – especially the one on psychopathology (Van Os, 2004; McKenzie et al., 2013) – has often been focused on the detrimental role of urban settings on human health, instead of speculating for an “opposite” effect of the benefits driven instead from the exposure to natural environments.

Visual examination of spectral power density patterns in the scalp topographic activity map showed that the difference in early-alphas was not limited to the central brain areas but extended also for the activity recorded over the occipital-central brain area. We performed exploratory analysis for this brain area, and we found similar patterns than those for the centralized alpha activity. However, in this case the difference between the conditions extended more toward the beta powers, and brain activity in response to nature and urban videos were very similar over all the frequency spectra, while different from the activity recorded for the control condition. A speculative explanation may be related to the very different visual content (fast lines and colors) of the neutral condition, which may be able to modulate in a very different manner the visual cortex compared to the other two more slowly changing and realistic experimental conditions.

Further visual inspection of the brainwaves pattern in graphical presentation of the power density × frequency analyses for the central cluster revealed that, alike early-alpha, a difference between the condition was also shown in late-theta spectral power density. This difference may be because of the higher perceived relaxation in response to the nature video condition. Several investigations have found that both alpha and theta power are associated to the activities of mindfulness and meditation (Aftanas and Golocheikine, 2001; Lagopoulos et al., 2009; Lomas et al., 2015), and therefore these brain waves may be both modulated in the same way during nature experiences in response to a relaxation and positive emotions. This effect seems to be in line with two recent studies [the lab-study of Sahni and Kumar (2020), and the real-life static exposure study of Chen et al. (2020)] showing that nature videos may modulate both alpha and theta power.

A recent study (Hopman et al., 2020) recorded resting state EEG brain activity before, during, and after a long, multi-day trip as exposure to natural environment. The researchers reported that posterior alpha power was significantly lower during the nature exposure when compared to measures taken before and after the visit to the natural environment. The authors did not analyze alpha waves over the central brain as such brain area was outside their a-priori brain region of interest, and therefore it is not possible from their data to understand the alpha modulation over the central brain. However, their midfrontal electrodes showed the same pattern reported from the posterior alpha power. These results show an opposite pattern compared to ours where instead exposure to natural settings increased alpha power over most of the brain, and especially as recorded over central and occipital brain areas. Such difference in results may be due to the different types of exposure to natural environments and different level of multi-sensory perception and integration. One should note that Hopman et al. (2020) did not include a similar multi-day trip to urban or other non-natural environment as a control condition. However, our lab experiment was more controlled than the experiment of Hopman et al. (2020), and the trip in natural settings intervention that they provided in their experiment may have brought with it several uncontrolled factors that may have affected physiological responses (e.g., familiarity with the visited environment, activities specific to the trip, moods/affections/stressors modulated by the trip, etc.). Therefore, it is not clear what explains the differences between our results [and similar results reported by earliest studies of Ulrich (1981) and Grassini et al. (2019)] and the results reported in Hopman et al. (2020).

The increase in alpha waves observed in our experiment suggests that the experience of nature video may be related to an increase activity of the inhibitory mechanism related to the activity of the thalamic-cortical circuit (Foxe and Snyder, 2011). In inhibiting attentional mechanisms, natural environments could be mediating stress reduction. The increase of theta activity over the mid-frontal brain areas during the vision of videos containing natural scenery may be related to increased cognitive control (Cooper et al., 2015). Cognitive control has been as well may have been related to stress (De Lissnyder et al., 2012). Both alpha and theta waves have been shown to be related to psychological states promoting relaxation as in the case of different types of meditation and mindfulness practices (Aftanas and Golocheikine, 2001; Lagopoulos et al., 2009; Lomas et al., 2015).

Statistical analyses for SCRs did not show any statistically significant differences between the studied conditions. Analyses for SCL showed that the lowest level of tonic activity was recorded during the urban videos, but the follow-up *t*-tests showed that the difference was statistically significant only in the comparison against the neutral condition. These findings are difficult to explain and are against the expected results (Alvarsson et al., 2010; Hedblom et al., 2019). However, recent studies have also reported heterogeneous findings regarding the modulation of EDA in response to natural environments (Browning et al., 2020). Some experimental characteristics (e.g., participants engagement, salience of the stimuli, etc.) may be modulating EDA in ways that were not experimentally controlled. Furthermore, it is possible that a more immersive stimulation (e.g., experiencing multi-sensory natural stimuli) may be more effective in modulating EDA, as compared with the videos in experimentally controlled settings.

Contrarily with some previous investigations [as reported in the review of Ohly et al. (2016)], the DSB task did not show an increase in performance after exposure to nature (but see Grassini et al., 2019). Nevertheless, in many of the previous studies revealing this effect the participants were exposed to a real and multi-sensorial experience of nature (e.g., exposure to real natural settings) of a long duration and generally connected with a physical activity in nature (e.g., walking, see Berman et al., 2008; Berman et al., 2012). The short duration of our exposure to natural stimuli, or the lack of a task involving physical activity during the exposure, may have affected the measurable effect of restoration in the DSB task. In our study, the relatively low level of immersion provided by digitalized videos, the short exposure to natural settings provided by the experimental setting, and the possible nuisance of wearing the EEG cap and EDA sensors, may have compromised the possibility of natural scenes to provide attention restoration. Furthermore, many of the previous studies showing attention restoration after nature exposure had given cognitive stressors to their participants prior to the experimental session, to give the possibility for cognitive restoration to baseline to happen. As no stressor was given to our participants, their baseline level of cognitive load may have been low enough and not allowing to detect eventual cognitive restorative effects of the exposure to nature videos. Also, the effect sizes reported in studies reporting DSB changes in response to environmental exposure are generally small, and therefore the present study was probably underpowered to detect these cognitive effects.

Additionally, the DSB used in our experiment was digitalized and visual, while the most used type of DSB in the literature has been auditory and with verbal responses (see, e.g., Berman et al., 2008, 2012). However, please note that previous studies have also used the type of DSB employed in the present investigation (see AuBuchon et al., 2015; Grassini et al., 2019). We cannot exclude that a different type of sensory stimulation or the different modality of administration of the test may influence the ability of the test to detect cognitive restoration.

The main limitation of the present study was that brain activity may be modulated by uncontrolled factors (e.g., colors, shapes, presence of moving items, etc.) systematically affecting one of the other experimental conditions. Furthermore, the “neutral” condition may arguably not be neutral but depicting man-made computerized objects which are processed similarly to urban ones (e.g., for some uncontrolled low-level visual similarities, such as sharp shapes).

## Conclusion

The results presented in our study, together with the ones presented in previous investigations (Ulrich, 1981; Grassini et al., 2019; Sahni and Kumar, 2020), showed that experiencing natural environments in various real or computerize forms produces measurable and reliable brain activity markers which are known to be related to cognitive and emotional restorative processes (Aftanas and Golocheikine, 2001; Lagopoulos et al., 2009; Baijal and Srinivasan, 2010). Therefore, they can be argued to promote restorative processes. The present study shows that even a type of nature visualization providing a relatively low-level of immersion – as videos – may be enough to promote some level of restoration. The presented results showed that brain activity – especially early-alpha waves recorded over the central brain – may represent a reliable index for nature-induced restoration, strengthening previous findings. On the other hand, contrarily to our expectations but in line with some recent literature, EDA-related indexes were not able to detect nature-induced restoration in our experimental setting. Differently to most previous studies on the topic, our investigation has the advantage of employing simultaneously both physiological and psychological methods to understand restorative processes during a lab-controlled exposure to videos, featuring both brain (EEG) and non-brain (EDA) physiological measures, and both self-assessment (relax rating of the videos), and behavioral test (DSB scores). In our knowledge, our study is one of the first ones attempting to understand if the positive effects reported in connection with natural experience may be driven by detrimental effect of urban settings or by proprieties of natural settings, our results supporting the latter alternative.

## Data Availability Statement

The raw data supporting the conclusions of this article will be made available by the authors, without undue reservation.

## Ethics Statement

The studies involving human participants were reviewed and approved by the Ethics Committee for Human Sciences at the University of Turku. The patients/participants provided their written informed consent to participate in this study.

## Author Contributions

SG developed the study idea, conceptualized the study design, responsible for the choice of the study methodology, developed the experimental paradigm, trained and supervised the laboratory assistants, responsible for the data analysis, data pre-processing scripts, and data curation, and had the main role in writing the manuscript draft. GS assisted the study planning and selection of experimental stimuli and responsible for the laboratory tests. MK assisted the development of the study, supervised the research project, and assisted the writing of the final version of the manuscript and the revisions of it. All authors contributed to the article and approved the submitted version.

## Conflict of Interest

The authors declare that the research was conducted in the absence of any commercial or financial relationships that could be construed as a potential conflict of interest.

## Publisher’s Note

All claims expressed in this article are solely those of the authors and do not necessarily represent those of their affiliated organizations, or those of the publisher, the editors and the reviewers. Any product that may be evaluated in this article, or claim that may be made by its manufacturer, is not guaranteed or endorsed by the publisher.

## References

[B1] Aftanas L. I. Golocheikine S. A. (2001). Human anterior and frontal midline theta and lower alpha reflect emotionally positive state and internalized attention: high-resolution EEG investigation of meditation. *Neurosci. Lett.* 310 57–60. 10.1016/s0304-3940(01)02094-8 11524157

[B2] Alvarsson J. J. Wiens S. Nilsson M. E. (2010). Stress recovery during exposure to nature sound and environmental noise. *Int. J. Environ. Res. Public Health* 7 1036–1046. 10.3390/ijerph7031036 20617017 PMC2872309

[B3] Annerstedt M. Jönsson P. Wallergård M. Johansson G. Karlson B. Grahn P. (2013). Inducing physiological stress recovery with sounds of nature in a virtual reality forest—results from a pilot study. *Physiol. Behav.* 118 240–250. 10.1016/j.physbeh.2013.05.023 23688947

[B4] Aspinall P. Mavros P. Coyne R. Roe J. (2015). The urban brain: analysing outdoor physical activity with mobile EEG. *Br. J. Sports Med.* 49 272–276. 10.1136/bjsports-2012-091877 23467965

[B5] AuBuchon A. M. Pisoni D. B. Kronenberger W. G. (2015). Short-term and working memory impairments in early-implanted, long-term cochlear implant users are independent of audibility and speech production. *Ear Hear.* 36:733. 10.1097/AUD.0000000000000189 26496666 PMC4621773

[B6] Baijal S. Srinivasan N. (2010). Theta activity and meditative states: spectral changes during concentrative meditation. *Cogn. Proc.* 11 31–38. 10.1007/s10339-009-0272-0 19626355

[B7] Bailey A. W. Allen G. Herndon J. Demastus C. (2018). Cognitive benefits of walking in natural versus built environments. *World Leisure J.* 60 293–305. 10.1080/16078055.2018.1445025

[B8] Benedek M. Kaernbach C. (2010). A continuous measure of phasic electrodermal activity. *J. Neurosci. Methods* 190 80–91. 10.1016/j.jneumeth.2010.04.028 20451556 PMC2892750

[B9] Benfield J. A. Taff B. D. Newman P. Smyth J. (2014). Natural sound facilitates mood recovery. *Ecopsychology* 6 183–188.

[B10] Berman M. G. Jonides J. Kaplan S. (2008). The cognitive benefits of interacting with nature. *Psychol. Sci.* 19 1207–1212. 10.1111/j.1467-9280.2008.02225.x 19121124

[B11] Berman M. G. Kross E. Krpan K. M. Askren M. K. Burson A. Deldin P. J. (2012). Interacting with nature improves cognition and affect for individuals with depression. *J. Affect. Disord.* 140 300–305. 10.1016/j.jad.2012.03.012 22464936 PMC3393816

[B12] Berto R. (2005). Exposure to restorative environments helps restore attentional capacity. *J. Environ. Psychol.* 25 249–259. 10.1016/j.jenvp.2005.07.001

[B13] Bevan M. D. Magill P. J. Terman D. Bolam J. P. Wilson C. J. (2002). Move to the rhythm: oscillations in the subthalamic nucleus–external globus pallidus network. *Trends Neurosci.* 25 525–531. 10.1016/s0166-2236(02)02235-x 12220881

[B14] Bigdely-Shamlo N. Mullen T. Kothe C. Su K. M. Robbins K. A. (2015). The PREP pipeline: standardized preprocessing for large-scale EEG analysis. *Front. Neuroinform.* 9:16. 10.3389/fninf.2015.00016 26150785 PMC4471356

[B15] Bratman G. N. Daily G. C. Levy B. J. Gross J. J. (2015). The benefits of nature experience: improved affect and cognition. *Landscape Urban Plann.* 138 41–50. 10.1016/j.landurbplan.2015.02.005

[B16] Brown D. K. Barton J. L. Gladwell V. F. (2013). Viewing nature scenes positively affects recovery of autonomic function following acute-mental stress. *Environ. Sci. Technol.* 47 5562–5569. 10.1021/es305019p 23590163 PMC3699874

[B17] Browning M. H. Mimnaugh K. J. van Riper C. J. Laurent H. K. LaValle S. M. (2020). Can simulated nature support mental health? comparing short, single-doses of 360-degree nature videos in virtual reality with the outdoors. *Front. Psychol.* 10:2667. 10.3389/fpsyg.2019.02667 32010003 PMC6974516

[B18] Cavanagh J. F. Shackman A. J. (2015). Frontal midline theta reflects anxiety and cognitive control: meta-analytic evidence. *J. Physiology-Paris* 109 3–15. 10.1016/j.jphysparis.2014.04.003 24787485 PMC4213310

[B19] Cavanagh J. F. Figueroa C. M. Cohen M. X. Frank M. J. (2012). Frontal theta reflects uncertainty and unexpectedness during exploration and exploitation. *Cereb. Cortex* 22 2575–2586. 10.1093/cercor/bhr332 22120491 PMC4296208

[B20] Cavanagh J. F. Frank M. J. Klein T. J. Allen J. J. (2010). Frontal theta links prediction errors to behavioral adaptation in reinforcement learning. *Neuroimage* 49 3198–3209. 10.1016/j.neuroimage.2009.11.080 19969093 PMC2818688

[B21] Chang C. Y. Chen P. K. (2005). Human response to window views and indoor plants in the workplace. *HortScience* 40 1354–1359. 10.21273/HORTSCI.40.5.1354 35581909

[B22] Chang C. Y. Hammitt W. E. Chen P. K. Machnik L. Su W. C. (2008). Psychophysiological responses and restorative values of natural environments in Taiwan. *Landscape Urban Plann.* 85 79–84. 10.1016/j.landurbplan.2007.09.010

[B23] Chen Z. He Y. Yu Y. (2020). Attention restoration during environmental exposure *via* alpha-theta oscillations and synchronization. *J. Environ. Psychol.* 68:101406. 10.1016/j.jenvp.2020.101406

[B24] Chiang Y. C. Li D. Jane H. A. (2017). Wild or tended nature? the effects of landscape location and vegetation density on physiological and psychological responses. *Landscape Urban Plann.* 167 72–83. 10.1016/j.landurbplan.2017.06.001

[B25] Choi Y. Kim M. Chun C. (2015). Measurement of occupants’ stress based on electroencephalograms (EEG) in twelve combined environments. *Building Environ.* 88 65–72. 10.1016/j.buildenv.2014.10.003

[B26] Cohen S. Spacapan S. (1978). The aftereffects of stress: an attentional interpretation. *Environ. Psychol. Nonverbal Behav.* 3 43–57. 10.1016/j.actpsy.2009.06.008 19632661

[B27] Cooper P. S. Wong A. S. Fulham W. R. Thienel R. Mansfield E. Michie P. T. (2015). Theta frontoparietal connectivity associated with proactive and reactive cognitive control processes. *Neuroimage* 108 354–363. 10.1016/j.neuroimage.2014.12.028 25528657

[B28] Coste C. P. Kleinschmidt A. (2016). Cingulo-opercular network activity maintains alertness. *Neuroimage* 128 264–272. 10.1016/j.neuroimage.2016.01.026 26801604

[B29] Critchley H. D. (2002). Electrodermal responses: what happens in the brain. *Neuroscientist* 8 132–142. 10.1177/107385840200800209 11954558

[B30] De Lissnyder E. Koster E. H. Goubert L. Onraedt T. Vanderhasselt M. A. De Raedt R. (2012). Cognitive control moderates the association between stress and rumination. *J. Behav. Ther. Exp. Psychiatry* 43 519–525. 10.1016/j.jbtep.2011.07.004 21813083

[B31] Delorme A. Makeig S. (2004). EEGLAB: an open source toolbox for analysis of single-trial EEG dynamics including independent component analysis. *J. Neurosci. Methods* 134 9–21. 15102499 10.1016/j.jneumeth.2003.10.009

[B32] Dosenbach N. U. Fair D. A. Cohen A. L. Schlaggar B. L. Petersen S. E. (2008). A dual-networks architecture of top-down control. *Trends Cogn. Sci.* 12 99–105. 10.1016/j.tics.2008.01.001 18262825 PMC3632449

[B33] Egner L. E. Sütterlin S. Calogiuri G. (2020). Proposing a framework for the restorative effects of nature through conditioning: conditioned restoration theory. *Int. J. Environ. Res. Public Health* 17:6792. 10.3390/ijerph17186792 32957693 PMC7558998

[B34] Elsadek M. Liu B. Xie J. (2020). Window view and relaxation: viewing green space from a high-rise estate improves urban dwellers’ wellbeing. *Urban Forestry Urban Green.* 55:126846.

[B35] Emfield A. G. Neider M. B. (2014). Evaluating visual and auditory contributions to the cognitive restoration effect. *Front. Psychol.* 5:548. 10.3389/fpsyg.2014.00548 24926279 PMC4046122

[B36] Engell T. Lorås H. W. Sigmundsson H. (2020). Window view of nature after brief exercise improves choice reaction time and heart rate restoration. *New Ideas Psychol.* 58:100781.

[B37] Fan Y. Duncan N. W. de Greck M. Northoff G. (2011). Is there a core neural network in empathy? an fMRI based quantitative meta-analysis. *Neurosci. Biobehav. Rev.* 35 903–911. 10.1016/j.neubiorev.2010.10.009 20974173

[B38] Foxe J. J. Snyder A. C. (2011). The role of alpha-band brain oscillations as a sensory suppression mechanism during selective attention. *Front. Psychol.* 2:154. 10.3389/fpsyg.2011.00154 21779269 PMC3132683

[B39] Gladwell V. F. Kuoppa P. Tarvainen M. P. Rogerson M. (2016). A lunchtime walk in nature enhances restoration of autonomic control during night-time sleep: results from a preliminary study. *Int. J. Environ. Res. Public Health* 13:280. 10.3390/ijerph13030280 26950138 PMC4808943

[B40] Gopal A. Clark E. Allgair A. D’Amato C. Furman M. Gansler D. A. (2013). Dorsal/ventral parcellation of the amygdala: relevance to impulsivity and aggression. *Psychiatry Res.: Neuroimaging* 211 24–30. 10.1016/j.pscychresns.2012.10.010 23352275

[B41] Grassini S. (2022). A systematic review and meta-analysis of nature walk as an intervention for anxiety and depression. *J. Clin. Med.* 11:1731. 10.3390/jcm11061731 35330055 PMC8953618

[B42] Grassini S. Revonsuo A. Castellotti S. Petrizzo I. Benedetti V. Koivisto M. (2019). Processing of natural scenery is associated with lower attentional and cognitive load compared with urban ones. *J. Environ. Psychol.* 62 1–11.

[B43] Haga A. Halin N. Holmgren M. Sörqvist P. (2016). Psychological restoration can depend on stimulus-source attribution: a challenge for the evolutionary account? *Front. Psychol.* 7:1831. 10.3389/fpsyg.2016.01831 27933011 PMC5120095

[B44] Hahn A. Stein P. Windischberger C. Weissenbacher A. Spindelegger C. Moser E. (2011). Reduced resting-state functional connectivity between amygdala and orbitofrontal cortex in social anxiety disorder. *Neuroimage* 56 881–889. 10.1016/j.neuroimage.2011.02.064 21356318

[B45] Hartig T. Böök A. Garvill J. Olsson T. Gärling T. (1996). Environmental influences on psychological restoration. *Scand. J. Psychol.* 37 378–393. 10.1111/j.1467-9450.1996.tb00670.x 8931393

[B46] Hartig T. Evans G. W. Jamner L. D. Davis D. S. Gärling T. (2003). Tracking restoration in natural and urban field settings. *J. Environ. Psychol.* 23 109–123.

[B47] Hartig T. Mitchell R. De Vries S. Frumkin H. (2014). Nature and health. *Annu. Rev. Public Health* 35 207–228. 24387090 10.1146/annurev-publhealth-032013-182443

[B48] Hedblom M. Gunnarsson B. Iravani B. Knez I. Schaefer M. Thorsson P. (2019). Reduction of physiological stress by urban green space in a multisensory virtual experiment. *Sci. Rep.* 9:10113. 10.1038/s41598-019-46099-7 31300656 PMC6625985

[B49] Hettinger N. (2012). “Nature restoration as a paradigm for the human relationship with nature,” in *Ethical Adaptation to Climate Change: Human Virtues of the Future*, eds Thompson A. Bendik-Keymer J. (Cambridge, MA: MIT Press), 27–46.

[B50] Hopman R. J. LoTemplio S. B. Scott E. E. McKinney T. L. Strayer D. L. (2020). Resting-state posterior alpha power changes with prolonged exposure in a natural environment. *Cogn. Res. Principles Implicat.* 5:51. 10.1186/s41235-020-00247-0 33108586 PMC7591649

[B51] Hunter M. D. Eickhoff S. B. Pheasant R. J. Douglas M. J. Watts G. R. Farrow T. F. (2010). The state of tranquility: subjective perception is shaped by contextual modulation of auditory connectivity. *Neuroimage* 53 611–618. 10.1016/j.neuroimage.2010.06.053 20600971

[B52] Jahncke H. Eriksson K. Naula S. (2015). The effects of auditive and visual settings on perceived restoration likelihood. *Noise Health* 17:1. 10.4103/1463-1741.149559 25599752 PMC4918645

[B53] Jones S. R. Kerr C. E. Wan Q. Pritchett D. L. Hämäläinen M. Moore C. I. (2010). Cued spatial attention drives functionally relevant modulation of the mu rhythm in primary somatosensory cortex. *J. Neurosci.* 30 13760–13765. 10.1523/JNEUROSCI.2969-10.2010 20943916 PMC2970512

[B54] Joye Y. Van den Berg A. (2011). Is love for green in our genes? a critical analysis of evolutionary assumptions in restorative environments research. *Urban Forestry Urban Green.* 10 261–268.

[B55] Joye Y. Steg L. Ünal A. B. Pals R. (2016). When complex is easy on the mind: internal repetition of visual information in complex objects is a source of perceptual fluency. *J. Exp. Psychol. Hum. Percept. Perform.* 42:103. 10.1037/xhp0000105 26322692

[B56] Kaplan R. Kaplan S. (1989). *The Experience of Nature: A Psychological Perspective.* Cambridge: Cambridge university press.

[B57] Kaplan S. (1995). The restorative benefits of nature: toward an integrative framework. *J. Environ. Psychol.* 15 169–182.

[B58] Kerr K. L. Avery J. A. Barcalow J. C. Moseman S. E. Bodurka J. Bellgowan P. S. (2015). Trait impulsivity is related to ventral ACC and amygdala activity during primary reward anticipation. *Soc. Cogn. Affect. Neurosci.* 10 36–42. 10.1093/scan/nsu023 24526181 PMC4994837

[B59] Kim G. W. Jeong G. W. Kim T. H. Baek H. S. Oh S. K. Kang H. K. (2010). Functional neuroanatomy associated with natural and urban scenic views in the human brain: 3.0 T functional MR imaging. *Korean J. Radiol.* 11 507–513. 10.3348/kjr.2010.11.5.507 20808693 PMC2930158

[B60] Kim H. G. Cheon E. J. Bai D. S. Lee Y. H. Koo B. H. (2018). Stress and heart rate variability: a meta-analysis and review of the literature. *Psychiatry Invest.* 15:235. 10.30773/pi.2017.08.17 29486547 PMC5900369

[B61] Kim M. J. Gee D. G. Loucks R. A. Davis F. C. Whalen P. J. (2011). Anxiety dissociates dorsal and ventral medial prefrontal cortex functional connectivity with the amygdala at rest. *Cereb. Cortex* 21 1667–1673. 10.1093/cercor/bhq237 21127016 PMC3116741

[B62] Korpela K. Kinnunen U. (2010). How is leisure time interacting with nature related to the need for recovery from work demands? testing multiple mediators. *Leisure Sci.* 33 1–14.

[B63] Korpela K. De Bloom J. Kinnunen U. (2015). From restorative environments to restoration in work. *Intell. Build. Int.* 7 215–223.

[B64] Kothe C. (2013). *The Artifact Subspace Reconstruction Method.* Available online at: https://sccn.ucsd.edu/githubwiki/files/asr-final-export.pdf (accessed January 25, 2022).

[B65] Lagopoulos J. Xu J. Rasmussen I. Vik A. Malhi G. S. Eliassen C. F. (2009). Increased theta and alpha EEG activity during nondirective meditation. *J. Alternative Complementary Med.* 15 1187–1192. 10.1089/acm.2009.0113 19922249

[B66] Lamm C. Decety J. Singer T. (2011). Meta-analytic evidence for common and distinct neural networks associated with directly experienced pain and empathy for pain. *Neuroimage* 54 2492–2502. 10.1016/j.neuroimage.2010.10.014 20946964

[B67] Laumann K. Gärling T. Stormark K. M. (2003). Selective attention and heart rate responses to natural and urban environments. *J. Environ. Psychol.* 23 125–134.

[B68] Lepore S. J. Evans G. W. (1996). “Coping with multiple stressors in the environment,” in *Handbook of Coping: Theory, Research, Applications*, eds Zeidner M. Endler N. S. (Hoboken, NJ: John Wiley & Sons), 350–377.

[B69] Leventhal D. K. Gage G. J. Schmidt R. Pettibone J. R. Case A. C. Berke J. D. (2012). Basal ganglia beta oscillations accompany cue utilization. *Neuron* 73 523–536. 10.1016/j.neuron.2011.11.032 22325204 PMC3463873

[B70] Lomas T. Ivtzan I. Fu C. H. (2015). A systematic review of the neurophysiology of mindfulness on EEG oscillations. *Neurosci. Biobehav. Rev.* 57 401–410. 10.1016/j.neubiorev.2015.09.018 26441373

[B71] McKenzie K. Murray A. Booth T. (2013). Do urban environments increase the risk of anxiety, depression and psychosis? an epidemiological study. *J. Affect. Disord.* 150 1019–1024. 10.1016/j.jad.2013.05.032 23742829

[B72] Miller K. J. Hermes D. Honey C. J. Hebb A. O. Ramsey N. F. Knight R. T. (2012). Human motor cortical activity is selectively phase-entrained on underlying rhythms. *PLoS One* 8:e1002655. 10.1371/journal.pcbi.1002655 22969416 PMC3435268

[B73] Miyakoshi M. (2021). *’s Preprocessing Pipeline.* Available online at: https://sccn.ucsd.edu/wiki/’s_preprocessing_pipeline (accessed September, 13, 2021).

[B74] Neuper C. Pfurtscheller G. (2001). Event-related dynamics of cortical rhythms: frequency-specific features and functional correlates. *Int. J. Psychophysiol.* 43 41–58. 10.1016/s0167-8760(01)00178-7 11742684

[B75] Norwood M. F. Lakhani A. Maujean A. Zeeman H. Creux O. Kendall E. (2019). Brain activity, underlying mood and the environment: a systematic review. *J. Environ. Psychol.* 65:101321.

[B76] Ohly H. White M. P. Wheeler B. W. Bethel A. Ukoumunne O. C. Nikolaou V. (2016). Attention restoration theory: a systematic review of the attention restoration potential of exposure to natural environments. *J. Toxicol. Environ. Health Part B* 19 305–343. 10.1080/10937404.2016.1196155 27668460

[B77] Park B. J. Tsunetsugu Y. Kasetani T. Hirano H. Kagawa T. Sato M. (2007). Physiological effects of shinrin-yoku (taking in the atmosphere of the forest)—using salivary cortisol and cerebral activity as indicators—. *J. Physiol. Anthropol.* 26 123–128. 10.2114/jpa2.26.123 17435354

[B78] Park B. J. Tsunetsugu Y. Kasetani T. Kagawa T. Miyazaki Y. (2010). The physiological effects of Shinrin-yoku (taking in the forest atmosphere or forest bathing): evidence from field experiments in 24 forests across Japan. *Environ. Health Prevent. Med.* 15 18–26. 10.1007/s12199-009-0086-9 19568835 PMC2793346

[B79] Piazza C. Miyakoshi M. Akalin-Acar Z. Cantiani C. Reni G. Bianchi A. M. (2016). “An automated function for identifying eeg independent components representing bilateral source activity,” in *Proceedings of the XIV Mediterranean Conference on Medical and Biological Engineering and Computing 2016*, (Cham: Springer). 10.1007/978-3-319-32703-7_22

[B80] Raanaas R. K. Patil G. Alve G. (2016). Patients’ recovery experiences of indoor plants and viewsof nature in a rehabilitation center. *Work* 53 45–55. 10.3233/WOR-152214 26684703

[B81] Ratcliffe E. Gatersleben B. Sowden P. T. (2013). Bird sounds and their contributions to perceived attention restoration and stress recovery. *J. Environ. Psychol.* 36 221–228. 10.1016/j.ctcp.2017.08.004 29122270

[B82] Roe J. J. Aspinall P. A. Mavros P. Coyne R. (2013). Engaging the brain: the impact of natural versus urban scenes using novel EEG methods in an experimental setting. *Environ. Sci.* 1 93–104.

[B83] Sadaghiani S. Scheeringa R. Lehongre K. Morillon B. Giraud A. L. Kleinschmidt A. (2010). Intrinsic connectivity networks, alpha oscillations, and tonic alertness: a simultaneous electroencephalography/functional magnetic resonance imaging study. *J. Neurosci.* 30 10243–10250. 10.1523/JNEUROSCI.1004-10.2010 20668207 PMC6633365

[B84] Sahni P. Kumar J. (2020). Effect of nature experience on fronto-parietal correlates of neurocognitive processes involved in directed attention: an ERP study. *Ann. Neurosci.* 27 136–147. 10.1177/0972753121990143 34556952 PMC8455014

[B85] Sherman M. A. Lee S. Law R. Haegens S. Thorn C. A. Hämäläinen M. S. (2016). Neural mechanisms of transient neocortical beta rhythms: converging evidence from humans, computational modeling, monkeys, and mice. *Proc. Natl. Acad. Sci. U S A.* 113 E4885–E4894. 10.1073/pnas.1604135113 27469163 PMC4995995

[B86] Siegel M. Donner T. H. Oostenveld R. Fries P. Engel A. K. (2008). Neuronal synchronization along the dorsal visual pathway reflects the focus of spatial attention. *Neuron* 60 709–719. 10.1016/j.neuron.2008.09.010 19038226

[B87] Sripada R. K. King A. P. Garfinkel S. N. Wang X. Sripada C. S. Welsh R. C. (2012). Altered resting-state amygdala functional connectivity in men with posttraumatic stress disorder. *J. Psychiatry Neurosci. JPN* 37:241. 10.1503/jpn.110069 22313617 PMC3380095

[B88] Stevenson M. P. Schilhab T. Bentsen P. (2018). Attention Restoration Theory II: a systematic review to clarify attention processes affected by exposure to natural environments. *J. Toxicol. Environ. Health Part B* 21 227–268. 10.1080/10937404.2018.1505571 30130463

[B89] Stigsdotter U. A. Grahn P. (2004). “A garden at your workplace may reduce stress,” in *Design & Health*, ed. Dilani A. (Stockholm: Research center for Design and Health).

[B90] Tyrväinen L. Ojala A. Korpela K. Lanki T. Tsunetsugu Y. Kagawa T. (2014). The influence of urban green environments on stress relief measures: a field experiment. *J. Environ. Psychol.* 38 1–9.

[B91] Tzoulas K. Korpela K. Venn S. Yli-Pelkonen V. Kaźmierczak A. Niemela J. (2007). Promoting ecosystem and human health in urban areas using green infrastructure: a literature review. *Landscape Urban Plann.* 81 167–178. 10.1016/j.scitotenv.2018.09.030 30321733

[B92] Ulrich R. S. (1979). Visual landscapes and psychological well-being. *Landscape Res.* 4 17–23. 10.1080/01426397908705892

[B93] Ulrich R. S. (1981). Natural versus urban scenes: some psychophysiological effects. *Environ. Behav.* 13 523–556. 10.1177/0013916581135001

[B94] Ulrich R. S. (1983). “Aesthetic and affective response to natural environment,” in *Behavior and the Natural Environment*, eds Altman I. Wohlwill J. F. (Boston, MA: Springer). 10.3389/fnhum.2021.676032

[B95] Ulrich R. S. Simons R. F. Losito B. D. Fiorito E. Miles M. A. Zelson M. (1991). Stress recovery during exposure to natural and urban environments. *J. Environ. Psychol.* 11 201–230. 10.1016/S0272-4944(05)80184-7

[B96] Van Os J. I. M. (2004). Does the urban environment cause psychosis? *Br. J. Psychiatry* 184 287–288. 10.1192/bjp.184.4.287 15056569

[B97] Veer I. M. Oei N. Y. Spinhoven P. van Buchem M. A. Elzinga B. M. Rombouts S. A. (2011). Beyond acute social stress: increased functional connectivity between amygdala and cortical midline structures. *NeuroImage* 57 1534–1541. 10.1016/j.neuroimage.2011.05.074 21664280

[B98] Vozzi A. Ronca V. Aricò P. Borghini G. Sciaraffa N. Cherubino P. (2021). The sample size matters: to what extent the participant reduction affects the outcomes of a neuroscientific research. a case-study in neuromarketing field. *Sensors* 21:6088. 10.3390/s21186088 34577294 PMC8473095

[B99] Welch P. (1967). The use of fast Fourier transform for the estimation of power spectra: a method based on time averaging over short, modified periodograms. *IEEE Trans. Audio Electroacoust.* 15 70–73. 10.1109/TAU.1967.1161901

[B100] Wilson E. O. (1984). *Biophilia.* Cambridge, MA: Harvard University Press.

[B101] Winkler I. Brandl S. Horn F. Waldburger E. Allefeld C. Tangermann M. (2014). Robust artifactual independent component classification for BCI practitioners. *J. Neural Eng.* 11:035013. 10.1088/1741-2560/11/3/035013 24836294

[B102] Yeo N. L. White M. P. Alcock I. Garside R. Dean S. G. Smalley A. J. (2020). What is the best way of delivering virtual nature for improving mood? an experimental comparison of high definition TV, 360 video, and computer generated virtual reality. *J. Environ. Psychol.* 72:101500. 10.1016/j.jenvp.2020.101500 33390641 PMC7772948

[B103] Yu C. P. Lee H. Y. Luo X. Y. (2018). The effect of virtual reality forest and urban environments on physiological and psychological responses. *Urban Forestry Urban Green.* 35 106–114.

